# Unveiling the Prognostic Power and Immune Landscape of MyD88 in Breast Cancer: an Integrative Bioinformatics and IHC Approach

**DOI:** 10.7150/jca.103403

**Published:** 2025-01-01

**Authors:** Xin Yu, Qinfang Zhang, Bei Li, Shengrong Sun, Juanjuan Li, Wenge Li

**Affiliations:** 1 Department of Breast and Thyroid Surgery, Renmin Hospital of Wuhan University, Wuhan, Hubei, P. R. China.; 2 Department of emergency, The 900 Hospital of the Joint Service Support Force of the People's Liberation Army of China, Fuzhou, Fujian, P. R. China.; 3 Department of Pathology, Renmin Hospital of Wuhan University, Wuhan, Hubei, P. R. China.; 4 Department of general surgery, Taikang Tongji (Wuhan) Hospital, Wuhan 430050, Hubei Province, P. R. China.; 5 Department of Oncology, Shanghai GoBroad Cancer Hospital, Shanghai, P. R. China.

**Keywords:** Breast cancer, MyD88, Prognosis, Cancer immunity, Bioinformatics

## Abstract

Breast cancer continues to be a significant global health challenge due to its heterogeneity and propensity for therapeutic resistance. The current tumor, node, and metastasis (TNM) staging and molecular classification systems are limited in capturing the full biological complexity of breast cancer. Myeloid differentiation primary response protein 88 (MyD88), a key adaptor protein in inflammatory signaling pathways, has been implicated in various oncogenic processes. This integrative study combines bulk RNA sequencing (RNA-seq), single-cell RNA sequencing (scRNA-seq), and immunohistochemistry (IHC) to explore the prognostic significance and immunological implications of MyD88 in breast cancer. We discovered that elevated MyD88 expression is associated with improved patient outcomes and is linked to the immunological landscape of breast cancer, including immune cell infiltration and the expression of immune checkpoint genes. Furthermore, our analysis indicates that MyD88 high-expressing tumors are more sensitive to chemotherapeutic agents, suggesting its potential as a predictive biomarker for therapeutic response. A nomogram incorporating MyD88 expression with other clinical variables was developed to estimate survival probabilities, enhancing the model's predictive accuracy. Our findings highlight MyD88 as a promising candidate for personalized medicine approaches in breast cancer, warranting further investigation into its mechanistic role and therapeutic potential.

## Introduction

Breast cancer remains a formidable adversary to global health, with its insidious nature and propensity for therapeutic resistance and recurrence post-treatment^1^. Despite the strides made in diagnostic precision and therapeutic innovation, the clinical landscape is riddled with variability in patient outcomes, even amongst those categorized under identical tumor stages and molecular subtypes. This underscores the limitations of the current tumor, node, and metastasis (TNM) staging and molecular classification systems, which fail to encapsulate the full spectrum of biological heterogeneity and prognostic intricacies within the breast cancer patient cohort^2^. The quest for enhanced predictive models and personalized treatment regimens has led to a renaissance in cancer research, with a spotlight on the exploration of novel biomarkers and cellular pathways^1^,^3^.

Myeloid differentiation primary response protein 88 (MyD88), a pivotal adaptor protein in the Toll-like receptor (TLR) and interleukin-1 receptor (IL-1R) signaling pathways^4^, has emerged as a promising candidate in the breast cancer research narrative. Its role in orchestrating inflammatory responses and cell survival signals positions it as a potential node of convergence for various oncogenic processes^5^.

This study aims to dissect the intricate relationship between MyD88 expression and its implications in the clinicopathological characteristics and prognostic outcomes of breast cancer. By employing an integrative approach that harnesses the power of bulk RNA sequencing (RNA-seq) and single-cell RNA sequencing (scRNA-seq), we intend to delineate the molecular underpinnings of MyD88's influence and to chart its interactions with the tumor microenvironment, particularly immune cell infiltration.

Through the application of immunohistochemistry (IHC), we validate the clinical relevance of MyD88 expression levels in breast cancer tissues, correlating elevated expression with better patient prognoses. Furthermore, leveraging bioinformatics tools, we endeavor to translate these findings into actionable insights that may refine clinical decision-making and catalyze the development of targeted therapeutic strategies. Our work represents a concerted effort to bridge the existing knowledge gaps and to contribute to the armamentarium against breast cancer, ultimately aiming to improve patient survival and quality of life.

## Materials and Methods

### Patients and specimens

TCGA-BRCA Cohort: The study involved 1091 patients diagnosed with breast cancer, and their RNA-seq data along with clinical records were acquired from The Cancer Genome Atlas (TCGA) via the Xena data portal at UCSC. Utilizing the TCGAbiolinks R package, somatic mutation datasets were meticulously retrieved and subsequently transformed into the Mutation Annotation Format (MAF) through the application of the maftools R package, thereby facilitating advanced downstream analysis.

In the case of the TCGA-BRCA cohorts, every individual with accessible transcriptomic sequence data and corresponding clinical details was incorporated into our analysis. This decision stemmed from the fact that there were no predefined rules for excluding subjects based on coexisting conditions or additional illnesses within the initial dataset.

IHC Cohort: Between February 2015 and August 2018, a comprehensive collection of formalin-fixed, paraffin-embedded tissue samples was meticulously assembled from a cohort of 100 female breast cancer patients at the Renmin Hospital of Wuhan University. Eligibility for this retrospective study was contingent upon a minimum 5-year follow-up period for the individuals involved. Prior to their inclusion, all participants had furnished their written consent, which had been duly vetted and approved by the Institutional Ethics Committee of Renmin Hospital at Wuhan University, as evidenced by the assigned protocol number WDRY2021-KS009. The study's primary endpoint was delineated by the incidence of either local recurrence or the emergence of distant metastasis. Regarding the IHC cohort, the eligibility requirements entailed: (i) histopathological verification of the disease, and (ii) the absence of any previous therapeutic interventions such as chemotherapy, radiotherapy, or immunotherapy prior to surgical excision. Conversely, individuals were excluded if they had: (i) concomitant health issues, (ii) substantial organ impairment, or (iii) the presence of other primary neoplasms elsewhere in the body.

The critical value for gene expression was determined using the survminer R package, which identified the optimal threshold for stratifying patients according to their gene expression levels.

### IHC

In accordance with the methodologies outlined in our preceding studies^6^, the IHC staining process was meticulously conducted through a series of standardized steps. Initially, the specimens underwent deparaffinization, followed by antigen retrieval to enhance the detection of target proteins. Subsequently, the endogenous peroxidase activity was neutralized by treatment with a 3% hydrogen peroxide solution for 25 minutes at room temperature, under conditions shielded from light. This was succeeded by a blockade with serum, utilizing 3% bovine serum albumin for 30 minutes at room temperature, to prevent nonspecific binding. The primary antibodies, specifically targeting MyD88 (ABCAM, ab133739, diluted at 1:100), were then applied and allowed to incubate overnight at 4°C. The final step involved conjugation with horseradish peroxidase (HRP) for a period of 50 minutes at room temperature, to facilitate the visualization of the antigen-antibody complex Visualization of the staining was achieved through the application of diaminobenzidine (DAB), with meticulous monitoring of the process using a microscope. Subsequently, the specimens were subjected to nuclear counterstaining by immersing them in a hematoxylin solution for an approximate duration of 3 minutes. The evaluation of the staining outcomes was predicated on the quantification and the degree of staining of the neoplastic cells that exhibited a positive reaction. The intensity of protein expression was stratified into four distinct grades: grade 0 for the absence of staining, grade 1 indicating weak staining characterized by a light brown hue, grade 2 for moderate staining with a brown color, and grade 3 denoting strong staining with a dark brown appearance. The protein staining score was ascertained through the application of a specific formula: the overall score equates to the product of the percentage score and the intensity score.

### Calculation of gene signature enrichment scores

In the analysis of transcriptomic data, we applied the gene set variation analysis (GSVA) method to anticipate the activity of specific pathways^7^. As previously documented^8^,^9^, an array of gene signatures relevant to cancer research was amassed, encompassing metabolic gene sets from the Kyoto Encyclopedia of Genes and Genomes (KEGG) database within MsigDB, along with drug-specific gene signatures. Some of these signatures are linked to pathways targeted by the immune system, while others forecast a patient's potential responsiveness to radiotherapy. A comprehensive list of all gene sets is provided in our previous work^8^,^9^. Additionally, the computation of scores for tumor-associated pathways across diverse samples was performed using the PROGENy software.

### Identification of differentially expressed genes (DEGs) and functional annotations

To identify genes with significant variation in expression levels, we utilized the Limma R package, employing stringent selection criteria that included a p-value threshold of less than 0.05 and a fold change (FC) ratio exceeding 1.5 for the detection of both upregulated and downregulated differentially expressed genes DEGs.

Subsequently, the ClusterProfiler R package was engaged to perform functional annotation and enrichment analysis of gene sets through the Gene Ontology (GO) and KEGG databases. Complementing this, gene set enrichment analysis (GSEA) was also executed with the ClusterProfiler package, with a particular emphasis on the significant enrichment of GO, KEGG, and Hallmark gene sets.

### Assessment of immune cell infiltration

For the purpose of dissecting the immunological landscape of breast cancer, we deployed CIBERSORT, a computational algorithm adept at quantifying the presence of 22 specific immune cell subtypes within the tumor microenvironment. Concurrently, leveraging the cancer immunity cycle gene sets delineated in the study by Xu *et al.* 10, we ascertained the enrichment scores of these gene sets through Gene Set Variation Analysis (GSVA). This facilitated the measurement of gene activity associated with diverse phases of the cancer immunity cycle in individual samples.

### Analysis of scRNA-seq

In this study, we employed scRNA-seq data, which includes annotations for cell clusters, in conjunction with paired bulk RNA-seq data sourced from the GEO database (GSE176078) encompassing 24 breast tumors. The unsupervised clustering of individual cells was accomplished using the Seurat R package. To guarantee the integrity of the scRNA-seq dataset, we implemented quality control measures, as previously described^8^,^11^. Additionally, we conducted an analysis focusing on intercellular communication networks, utilizing the iTalk R package.

### Prediction of chemotherapy response

The determination of half-maximal inhibitory concentration (IC50) for prevalent chemotherapeutic agents was conducted through the "pRRophetic" R package.

### Development of the nomogram associated with MyD88

To identify potential autonomous prognostic indicators, a multivariate Cox analysis was executed on MyD88 expression and clinical variables. Subsequently, using regplot software, we formulated a nomogram linked to MyD88, encompassing age, stage, Pam50 subtypes, and MyD88 expression as variables. This nomogram facilitates the estimation of survival probabilities for breast cancer patients by incorporating age, stage, Pam50 subtypes, and MyD88 expression.

### Statistical analysis

In our quest to elucidate the interconnections among a spectrum of variables, we applied Pearson correlation analysis to quantify their relationships. For continuous variables that conformed to a Gaussian distribution, we employed a t-test to discern differences between two distinct cohorts. When it came to delineating disparities among multiple groups, the Kruskal-Wallis test was the analytical tool of choice. To scrutinize statistically significant variations within the subsets of our datasets, the log-rank test was engaged, and survival trajectories were delineated employing the Kaplan-Meier (KM) method. SangerBox^12^ and R 4.0.0 were utilized for all statistical analyses. The p-values obtained were two-tailed, with significance levels set at less than 0.05.

## Results

### The expression of MyD88 in breast cancer

**Figure 1:**
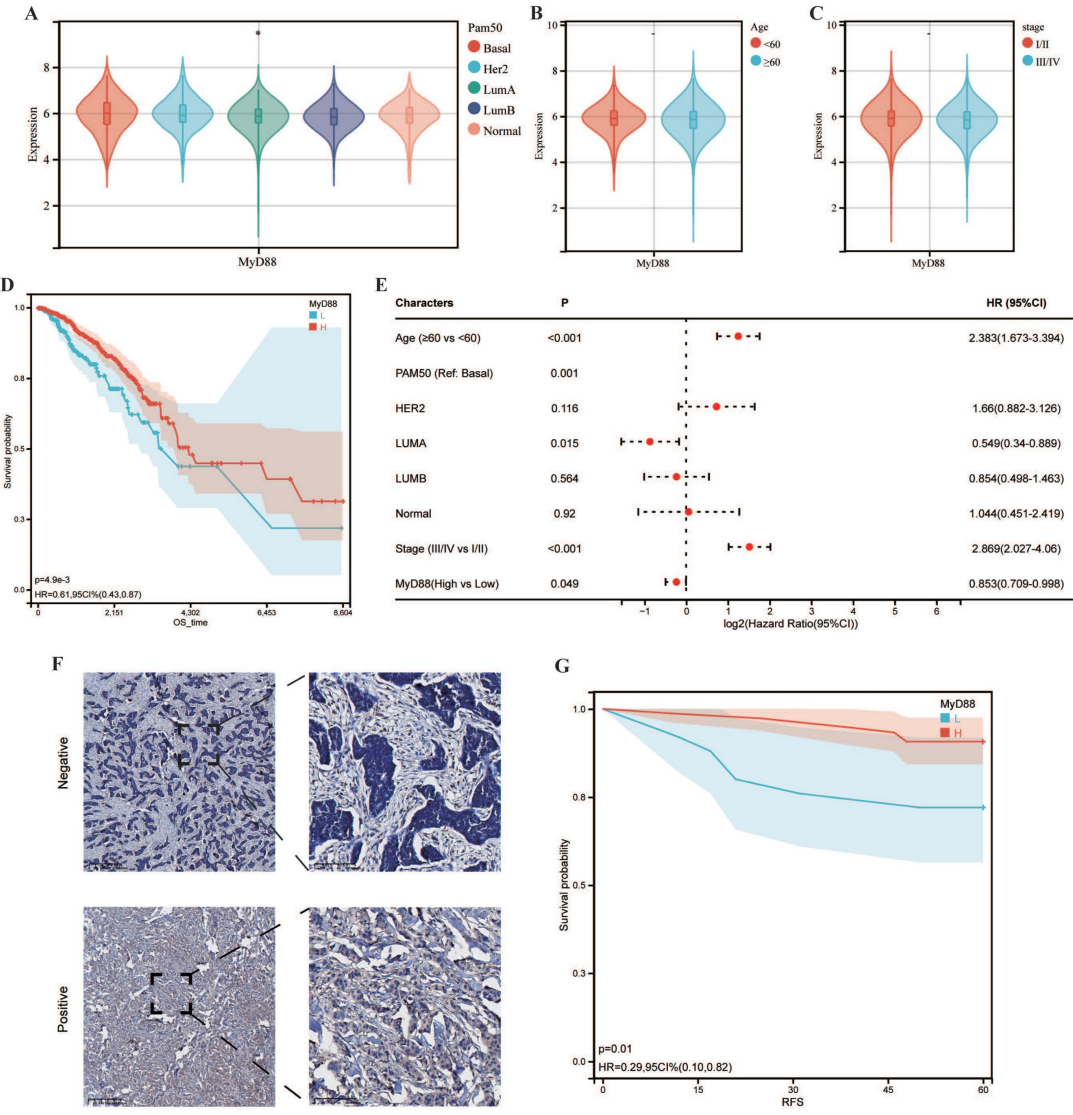
** MyD88 transcriptional profile and prognostic significance in breast cancer.** (A-C) Transcriptional expression of MyD88 stratified by (A) Pam50 subtypes, (B)Age, and (C) stage. (D-E) Kaplan-Meier (D) and Cox regression (E) analyses evaluating the prognostic value of MyD88 in overall survival (OS) using TCGA-cohort data.(F) Representative immunofluorescence staining images illustrating MyD88 expression. (G) RFS curves for MyD88 in the IHC-cohort. Statistical significance denoted as *p < 0.05, **p < 0.01, ***p < 0.001, ****p < 0.0001.

Within the scope of this research, we conducted an inquiry into the relationship between MyD88 expression levels and the clinical manifestations of breast cancer. Commencing with a stratification of breast cancer patients by their molecular subtypes, we discerned a pronounced elevation in MyD88 expression within the basal and normal-like subtypes when juxtaposed with the Luminal A, Luminal B, and HER2-enriched subtypes (Figure 1A). A noteworthy observation was the absence of a significant disparity in MyD88 expression levels across patients categorized by varying stages and age brackets (Fig. 1B, 1C). To investigate the prognostic value of MyD88, we divided the TCGA-cohort patients into high and low expression groups. The low MyD88 group exhibited less favorable clinical outcomes according to KM analysis (Fig. 1D). Furthermore, in breast cancer patients, MyD88 emerged as a noteworthy independent prognostic predictor, as evidenced by multivariate Cox regression analysis (Fig. 1E). To validate our findings, we performed IHC on 100 breast cancer patients, confirming that MyD88 primarily expressed in epithelial cells, with a diffuse cytoplasmic distribution (Fig. 1F). Consistently, long recurrence-free survival (RFS) was associated with elevated MyD88 expression, as illustrated by KM analysis (Fig. 1G). These results suggest that MyD88 may serve as a prognostic biomarker in breast cancer.

### Identification of DEGs and functional annotations

**Figure 2:**
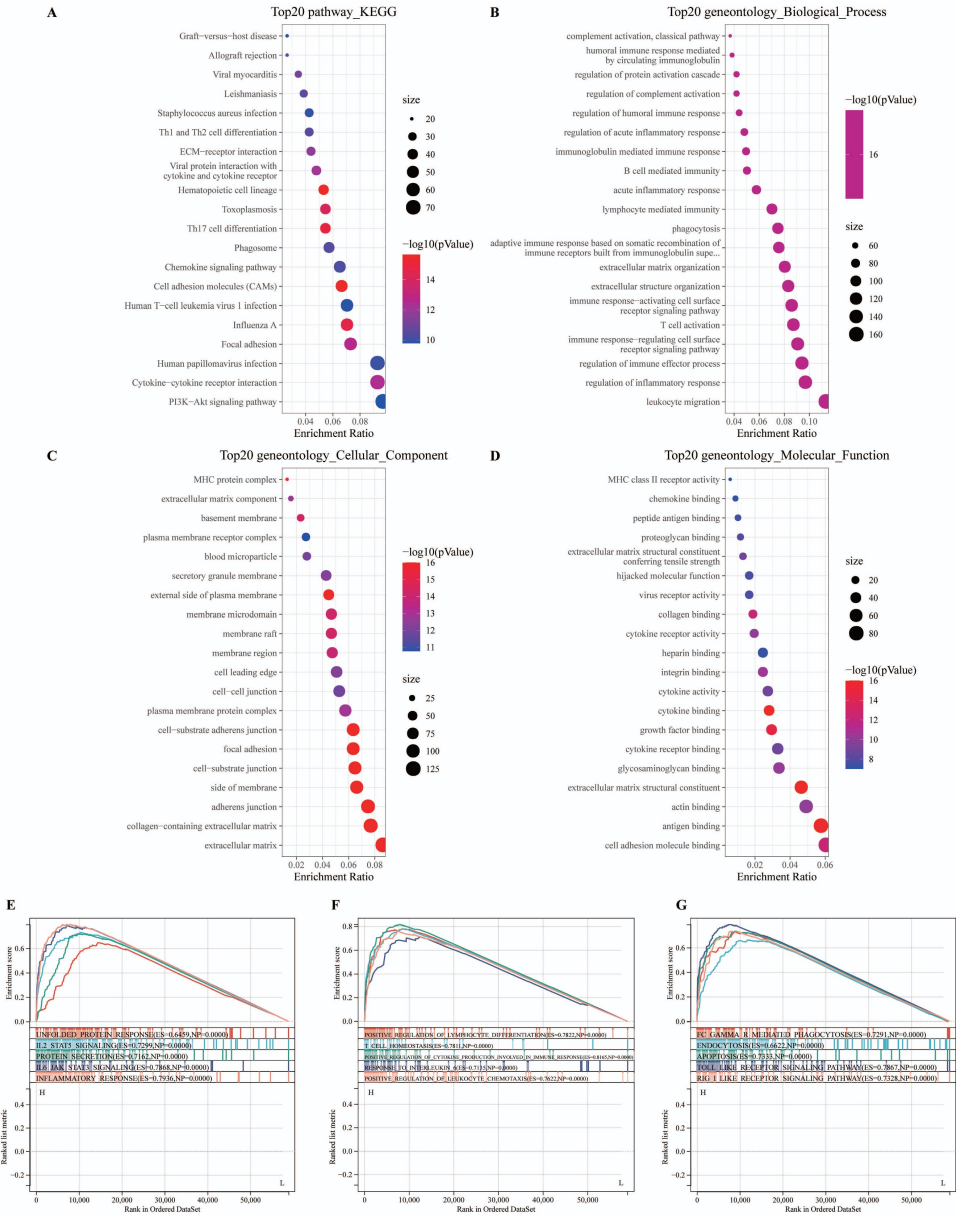
** Functional annotation of MyD88.** (A-C) Enrichment analysis of differentially expressed genes (DEGs) was conducted across various databases: (A) KEGG for pathway mapping, (B) GO-BP for biological process categorization, and (C) GO-CC for cellular component identification, along with (D) GO-MF for molecular function analysis. (E-G) Functional profiling of MyD88 was performed using Gene Set Enrichment Analysis (GSEA) with gene sets derived from (E) KEGG pathways, (F) GO pathways, and (G) the hallmark gene pathways.

Upon the delineation of differentially expressed genes (DEGs), our analysis indicated a substantial upregulation of 1680 genes, in contrast to the downregulation of merely 4 genes within the cohort exhibiting elevated MyD88 expression levels (Supplementary Fig. S1 A, B). To gain deeper insights into the biological functions of these DEGs, we performed pathway analysis. The results revealed that the upregulated DEGs were predominantly associated with "Graft-versus-host disease" , "Allograft rejection", and the "Viral myocarditis" signaling pathway in diabetic complications" within the KEGG pathways (Fig. 2A). GO analysis demonstrated significant enrichment of these genes in processes such as "complement activation, classical pathway," "humoral immune response mediated by circulating immunoglobulin," and "regulation of protein activation cascade" in the biological process (BP) category (Fig. 2B), "MHC protein complex," "extracellular matrix component," and " basement membrane" (CC) category (Fig. 2C), and "MHC class II receptor activity," "chemokine binding," and "peptide antigen binding" in the molecular function (MF) category (Fig. 2D).

To further explore the connection between MyD88 and breast cancer, a GSEA was conducted. The results revealed that patients with elevated MyD88 expression exhibited higher enrichment of Hallmark pathways such as "UNFOLDED_PROTEIN_RESPONSE,""IL2_STAT5_SIGNALING","PROTEIN_SECRETION," (Fig. 2E), as well as KEGG gene sets including "Adherens junction," "Arrhythmogenic right ventricular cardiomyopathy ARVC," and "Endocytosis" (Fig. 2F). Additionally, GO gene sets such as "Negative regulation of gluconeogenesis," "Negative regulation of endoplasmic reticulum calcium ion concentration," and "Regulation of cardiac muscle adaptation" were enriched in patients with elevated MyD88 expression (Fig. 2G). Collectively, these observations underscore the pivotal role that MyD88 plays in the oncogenic mechanisms linked to breast cancer development.

### The association of MyD88 with carcinogenic pathways and gene mutations

**Figure 3:**
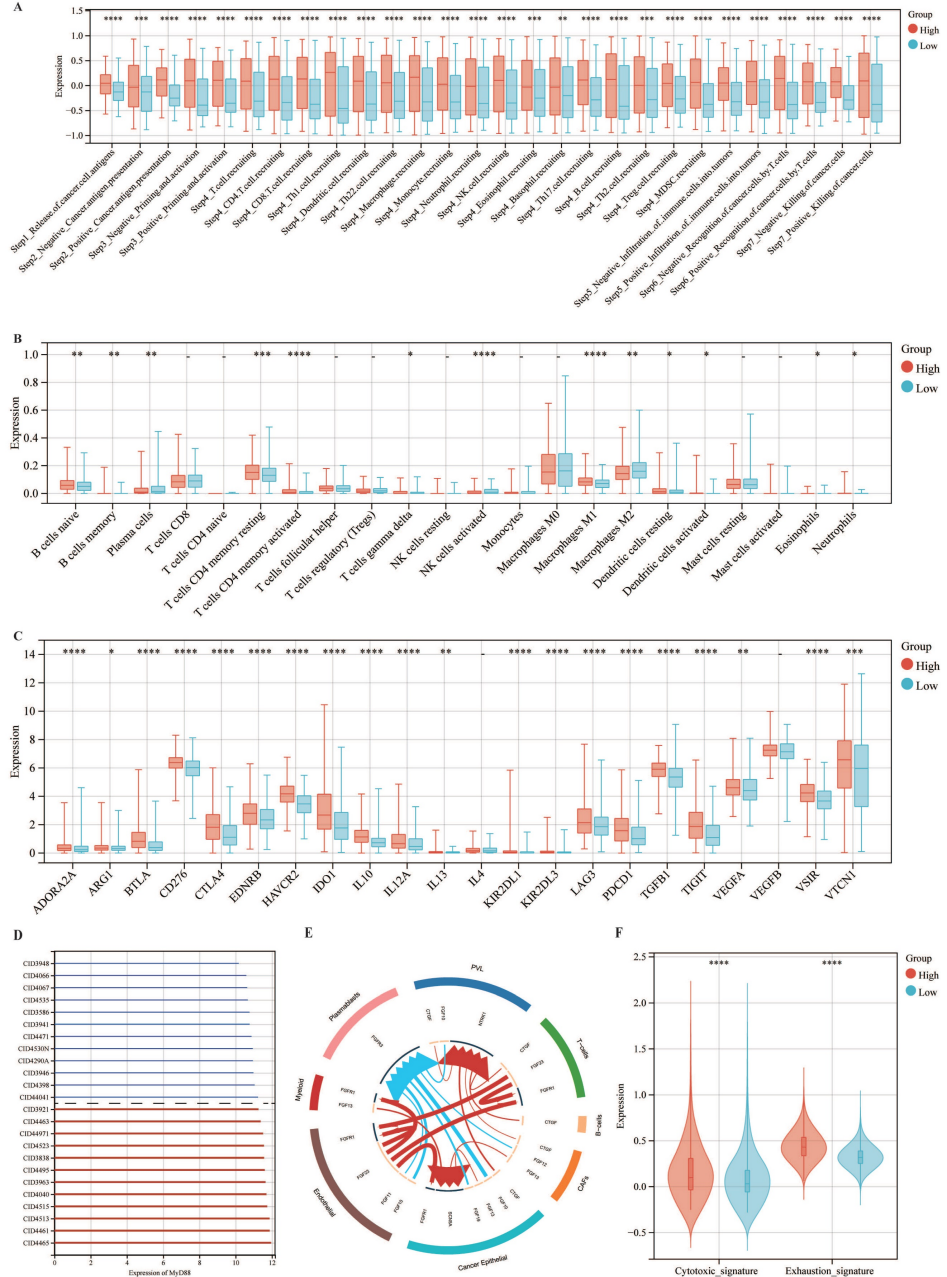
** The association of MyD88 with carcinogenic pathways and gene mutations.** (A-C) Differential expression of (A) immune cycle score, (B) immune cells, and (C) immune checkpoints in high and low MyD88 groups. (D) MyD88 distribution in 24 breast cancer samples (divided into 2 patterns). (E) Circos plots illustrating ligand-receptor interactions with significant expression differences in the TIME. (F) Differences in cytotoxic and exhausted T cell scores between high and low MyD88 groups. Significance denoted as “-” p > 0.05, “*” p < 0.05, “**” p < 0.01, “***” p < 0.001, “****” p < 0.0001.

Subsequently, we explored the association between MyD88 and the carcinogenic pathways implicated in breast cancer. Our findings revealed that a majority of carcinogenic pathways, including JAK-STAT, hypoxia, NFkB, TNFa, TGFb, MAPK, p53, Estrogen, Androgen, PI3K, TGFb, Trail, WNT and VEGF, exhibited upregulation in the high MyD88 expression group (Fig. 3A, B). Analysis of the mutational landscape disclosed a heightened overall mutation frequency within the group characterized by high MyD88 expression, with particularly notable surges in the mutation rates of the SPTA1 and CCDC168 genes. In contrast, the mutation rate significantly increased in the low MyD88 expression group for the gene TTN (Fig. 3C). An exhaustive examination of MyD88's impact within the tumor immune microenvironment (TIME) was executed by integrating scRNA-seq data with corresponding bulk RNA sequencing data sourced from a cohort of 24 breast cancer patients, as cataloged in the GEO under the accession number GSE176078.Based on the stratification of MyD88 expression levels, the study population of 24 individuals was categorized into two distinct groups: one with high MyD88 expression and another with low MyD88 expression, using the median expression value as the threshold for differentiation (Fig. 3D).Within the T cell Cytotoxic and Exhaustion signatures, the group exhibiting elevated MyD88 expression demonstrated higher levels compared to the group with reduced expression. T cells characterized by high MyD88 expression levels were associated with increased cytotoxicity and exhaustion scores relative to those with lower expression (Fig. 3F).

### Correlations between MyD88 and metabolism pathways

**Figure 4:**
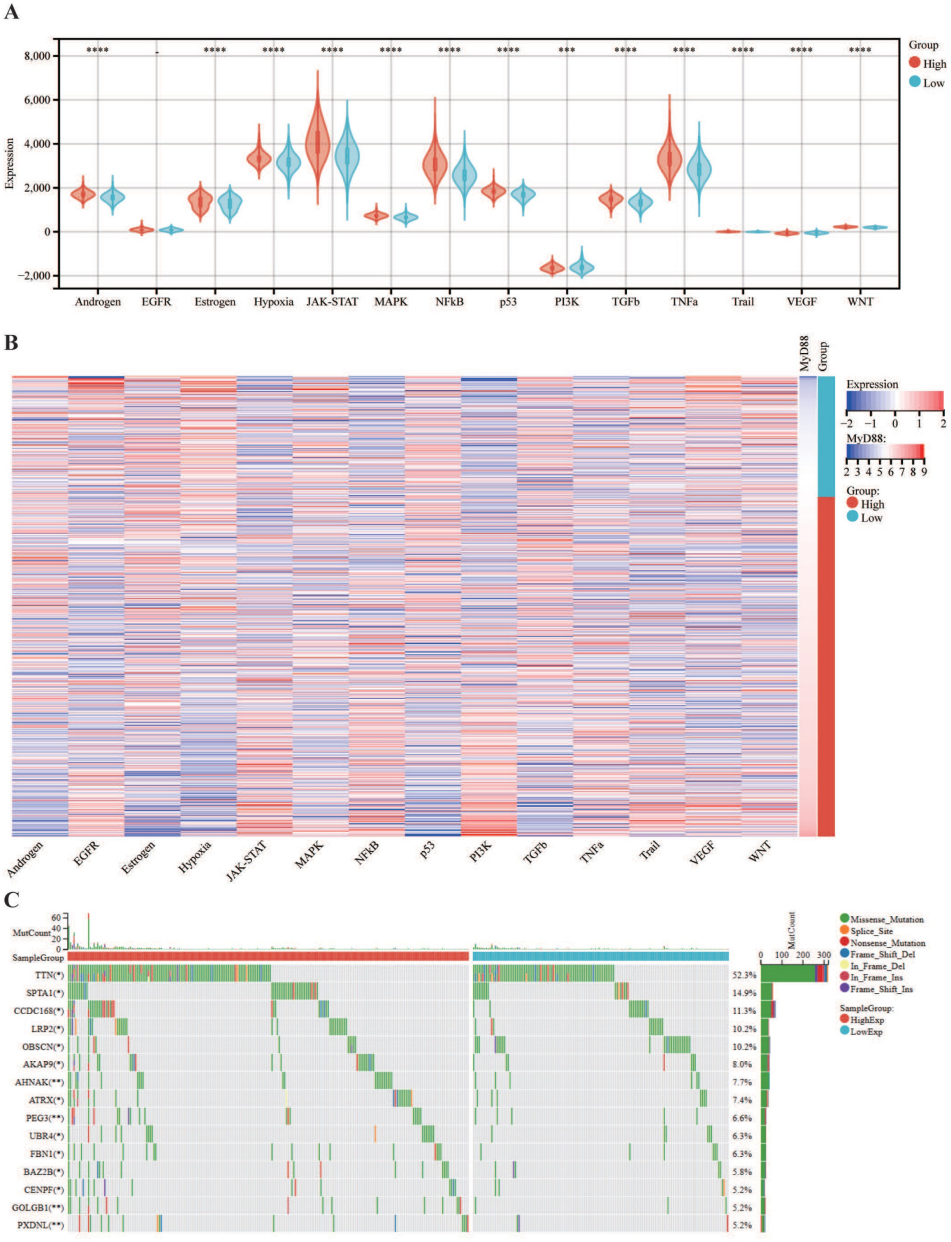
** Carcinogenic Pathways and Mutation Landscape Comparison.** (A-B) Differential expression (A) and heatmap (B) of carcinogenic pathways in groups with high and low MyD88. (C) Mutation landscape comparison between high and low MyD88 groups. Significance denoted as “-” p > 0.05, “*” p < 0.05, “**” p < 0.01, “***” p < 0.001, “****” p < 0.0001.

In the 70 KEGG metabolic pathways, the MyD88 high expression group exhibited upregulation in 52 pathways, including the majority of pathways involved in amino acid metabolism, carbohydrate metabolism, lipid metabolism, and nucleic acid metabolism, which are well-recognized as oncogenic metabolic pathways (Fig. 4). However, within the MyD88 high expression group, the pathways of taurine and hypotaurine metabolism^13^ and oxidative phosphorylation^14^ were downregulated. These pathways are associated with the suppression of the tumor immune microenvironment.

### The expression of MyD88 association with TIME

**Figure 5:**
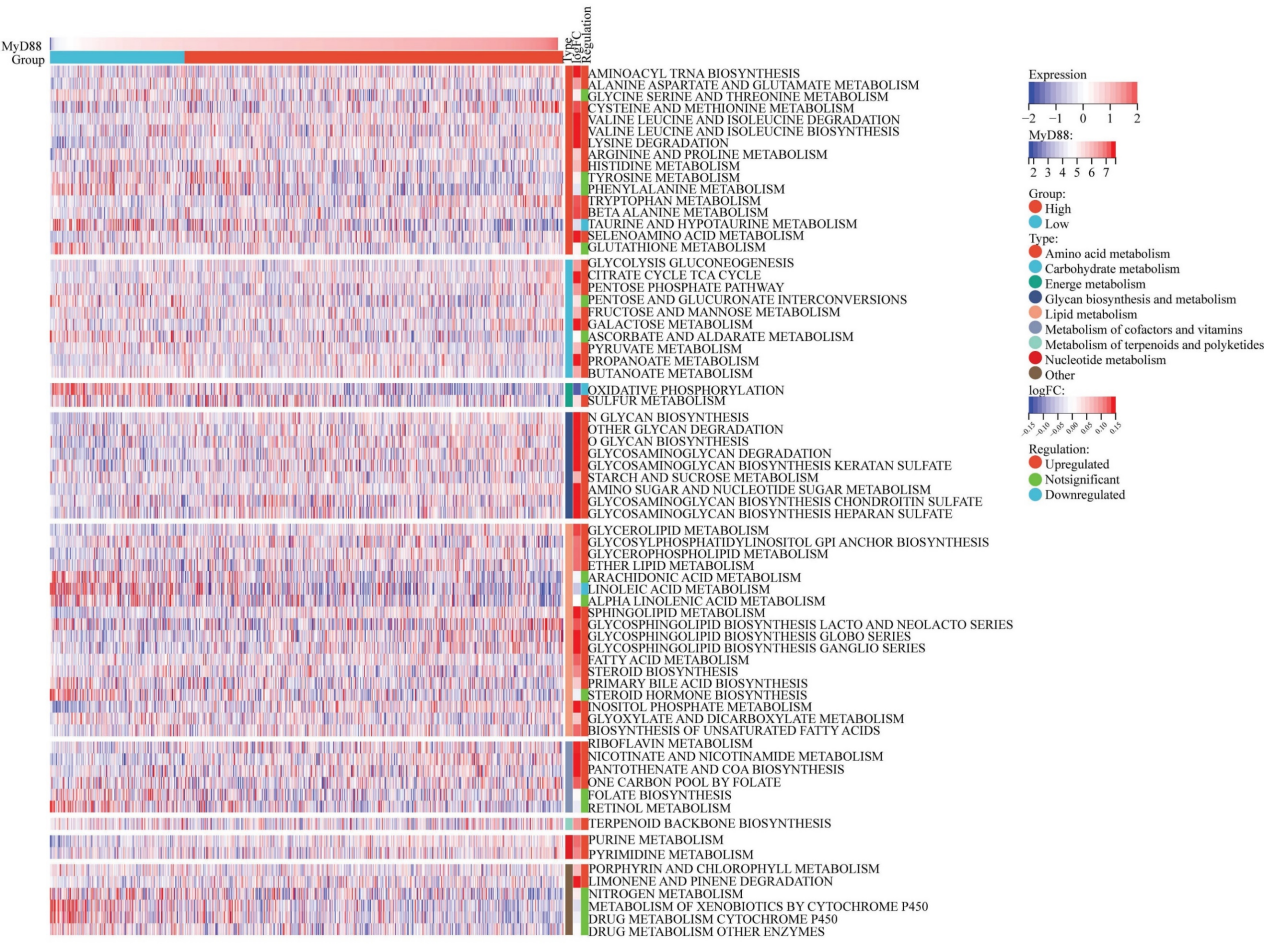
** A heatmap is featured, illustrating the correlation analysis between MyD88 expression levels and breast cancer metabolism.** The visual representation delineates the expression patterns of metabolic signatures, contrasting the profiles observed in patients with elevated versus reduced MyD88.

Assessing the influence of MyD88 on the TIME, our study encompassed an appraisal of the efficacy of cancer immune responses, an examination of immune cell infiltration, and a dissection of the expression profiles of immune checkpoint genes (Fig. 5). Notably, in patients with high MyD88 expression, all immunoinfiltrate positive and negative regulatory scores increased (Fig. 5A). Similarly, CIBERSORT estimates of immune cell infiltration showed that patients with high MyD88 expression had more cancer-suppressing immune cells, such as M1 macrophages, and fewer cancer-promoting immune cells, such as M2 macrophages (Fig. 5B). Furthermore, an upregulation of the majority of immune checkpoint-associated genes was observed in individuals with elevated MyD88 expression levels, encompassing notable genes such as CD276, PDCD1, CTLA4, and LAG3 (Fig. 5C). The above results indicated that there was a relatively strong immune infiltration and immune escape antagonistic process in patients with high expression of MyD88.

### Potential of the expression of MyD88 association for predicting therapeutic opportunities

**Figure 6:**
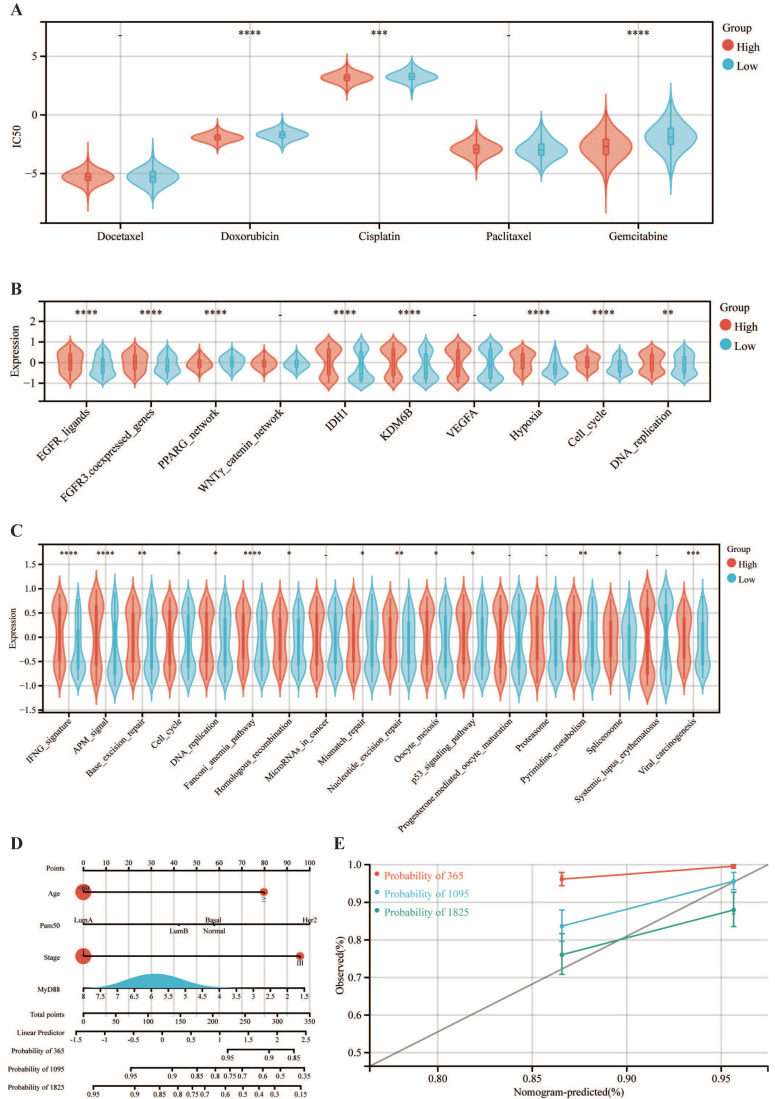
** Correlation of MyD88 in the treatment of breast cancer and establishment of a nomogram.** (A-C) Differential expression of (A) IC50 of chemotherapy drugs, (B) targeted therapy pathway characteristics, and (C) immunotherapy-predicted pathways in high and low MyD88 groups. (D) Nomogram predicting overall survival probability using age, Pam50 subtypes, tumor stage, and MyD88 expression. (E) Calibration curves validating prediction accuracy for 1-year (red), 3-year (blue), and 5-year (green) survival predictions. Significance denoted as “-” p > 0.05, “*” p < 0.05, “**” p < 0.01, “***” p < 0.001, “****” p < 0.0001.

Pharmacological sensitivity profiling revealed that the cohort with elevated MyD88 expression exhibited heightened sensitivity to chemotherapeutic agents, including doxorubicin, cisplatin, and gemcitabine (Fig. 6A). Analyses indicate that the MyD88 high-expressing group exhibits upregulated expression in pathways associated with EGFR ligands, FGFR3 coexpressed genes, IDH1, KDM6B, VEGFA, hypoxia, and DNA replication, suggesting the feasibility of targeting these pathways with therapeutic agents (Fig. 6B). Further in-depth explorations into the correlation with immune pathways have uncovered a robust positive link between heightened MyD88 expression and the activation of immune-related pathways, with the most pronounced correlations detected within the IFNG signature, antigen processing and presentation signaling, and the Fanconi anemia pathway (Fig. 6C).

### Development and validation of MyD88-related nomogram

We have crafted a nomogram that encapsulates a range of clinicopathological variables to prognosticate patient survival with clinical applicability. This predictive tool was grounded in the robust statistical framework provided by Cox proportional hazards analysis, allowing us to delineate overall survival (OS) trajectories (Fig. 6D). Notably, the incorporation of MyD88 expression into our model significantly enhanced its predictive accuracy, as evidenced by the calibration curves that corroborate the model's precision in estimating survival probabilities. (Fig. 6E).

## Discussion

In our research, we explored the expression profiles and clinical relevance of MyD88 in breast cancer, revealing its complex contributions to the disease's advancement. Our findings elucidate the intricate connections between MyD88 and various clinical characteristics, prognostic outcomes, metabolic pathways, immune microenvironments, and potential therapeutic targets.

MyD88, a critical adaptor protein in the signaling pathways of TLRs and IL-1Rs, is known to orchestrate inflammatory responses and cell survival signals. The MyD88 protein serves as a pivotal conduit in inflammatory signaling pathways, bridging IL-1R or TLR family receptors to the IL-1R-associated kinase (IRAK) family of kinases through homotypic protein interactions. Once activated, these IRAK kinases initiate a cascade of downstream effects, encompassing the stimulation of nuclear factor-kappa B (NFκB), mitogen-activated protein kinases (MAPKs), and activator protein 1 (AP-1), thereby cementing MyD88's role as a central hub in the inflammatory response^15^.A number of deactivating mutations within the MyD88 gene have been pinpointed in individuals experiencing recurrent infections caused by pyogenic bacteria^16^. Its dual role in promoting or suppressing tumorigenesis has been previously suggested, with its exact function depending on the cellular context and cancer type. Integral to the innate immune response, MyD88's activation is instrumental in driving the synthesis of inflammatory cytokines and modulating the tumor microenvironment by facilitating the infiltration, polarization, and evasion of immune cells. Furthermore, aberrant MyD88 signaling is implicated in the proliferation and metastasis of tumor cells, processes that are significantly correlated with an adverse patient prognosis^17^.

The advent of high-throughput technologies such as RNA sequencing and bioinformatics has propelled the identification of novel biomarkers and our understanding of their roles in cancer biology. Our integrative analysis of bulk RNA-seq and scRNA-seq data, coupled with IHC validation, revealed that elevated MyD88 expression is associated with improved patient prognoses. This association underscores MyD88's potential as a prognostic biomarker in breast cancer.

Our investigation also explored the correlation between MyD88 and carcinogenic pathways, revealing its involvement in key signaling cascades that contribute to tumor progression. Significantly, our findings indicate that the impact of MyD88 is not confined to immune responses but also permeates into the metabolic processes of the tumor. Elevated levels of MyD88 expression are associated with heightened activity in key metabolic pathways that are essential for cancer metabolism, including those involved in the turnover of amino acids, carbohydrates, lipids, and nucleic acids.

Furthermore, our analysis of the immune microenvironment indicated that MyD88 expression is linked to the immunological landscape of breast cancer. Individuals exhibiting heightened MyD88 expression displayed a tumor microenvironment that fosters immune activity, marked by an upsurge in immune cells that suppress cancer, such as M1 macrophages, and a concurrent reduction in those that may promote cancer development, including M2 macrophages. The MyD88 molecule is capable of triggering the expression of a spectrum of chemokines and cytokines pivotal to immune responses, including tumor necrosis factor alpha (TNF-α), interleukin-6 (IL-6), inducible nitric oxide synthase (iNOS), cyclooxygenase-2 (COX-2), type III interferons (IFN-IIIs), and interleukin-10 (IL-10)^18^,^19^. Advanced research has increasingly revealed the active role of inflammation in carcinogenesis, with findings underscoring its facilitation of tumor growth and the suppression of immune responses. This finding was corroborated by scRNA-seq data, which showed higher cytotoxic and exhaustion scores in T cells from patients with high MyD88 expression^20^.

The therapeutic implications of our findings are also noteworthy. We discovered that the high MyD88 expression group displayed heightened sensitivity to chemotherapeutic agents such as doxorubicin, cisplatin, and gemcitabine. This suggests that MyD88 expression levels could be leveraged to predict therapeutic responses and guide treatment decisions.

MyD88, a key adaptor protein in breast cancer, holds immense clinical potential. Studies indicate that high MyD88 expression is associated with improved prognosis, making it a valuable prognostic marker. Furthermore, it can guide treatment decisions by predicting chemotherapy sensitivity, identifying potential targets for targeted therapy, and assessing response to immunotherapy. Future research exploring its mechanism of action and conducting clinical validation is crucial to fully harness its therapeutic potential and improve outcomes for breast cancer patients.

Despite these findings, our study is not without limitations. Firstly, the use of patient samples from a single clinical center may introduce some biases. Secondly, our study is retrospective in nature, which introduces some limitations such as potential confounding factors in determining causality and the need for further validation through *in vitro* and *in vivo* experiments. Overall, further research is needed to elucidate the exact function and mechanism of action of MyD88. Our study, while contingent upon digital databases and clinical samples that warrant further validation through experimental assays both *in vitro* and *in vivo*, establishes MyD88 as a critical component in the etiology of breast cancer. It holds substantial promise as a prognostic indicator and a potential therapeutic intervention target. Subsequent research endeavors should be directed towards elucidating the intricate mechanisms underlying MyD88's role in breast cancer progression, as well as harnessing its potential for devising innovative, precision-targeted therapeutic approaches.

## Supplementary Material

Supplementary figure.

## Abbreviations

AP-1: activator protein 1
COX-2: Cyclooxygenase 2
DAB: Diaminobenzidine
DEGs: Differentially Expressed Genes
FC: Fold Change
GO: Gene Ontology
GSEA: Gene Set Enrichment Analysis
GSVA: Gene Set Variation Analysis
HRP: Horseradish Peroxidase
IC50: Half-Maximal Inhibitory Concentration
IFN-IIIs: Type III IFNs
IHC: Immunohistochemistry
IL-10: Interleukin-10
IL-1R: Interleukin-1 Receptor
IL-6: Interleukin-6
iNOS: Inducible Nitric Oxide Synthase
IRAK: IL-1R-Associated Kinase
KEGG: Kyoto Encyclopedia of Genes and Genomes
KM: Kaplan-Meier
MAF: Mutation Annotation Format
MAPKs: mitogen-activated protein kinases
MyD88: Myeloid Differentiation Primary Response Protein 88
NFκB: Nuclear Factor-Kappa B
OS: Overall Survival
RFS: Recurrence-Free Survival
RNA-seq: RNA Sequencing
scRNA-seq: Single-Cell RNA Sequencing
TCGA: The Cancer Genome Atlas
TIME: Tumor Immune Microenvironment
TLR: Toll-Like Receptor
TNF-α: Tumor Necrosis Factor Α
TNM: Tumor, Node, and Metastasis
