# Supplementary material for: Unveiling the Prognostic Power and Immune Landscape of MyD88 in Breast Cancer: an Integrative Bioinformatics and IHC Approach
Source: J Cancer. 2025 Jan 1;16(1):201–13. doi: 10.7150/jca.103403 (PMC11660130; doi:10.7150/jca.103403)
Supplement: Supplementary file 1 — Supplementary figure. [file jcav16p0201s1.pdf]

Figure S1: Identification of DEGs and functional annotations.

(A)Among the differential genes, 1680 genes were up-regulated and 4 genes were down-regulated,.

(B)Differential gene heat map.

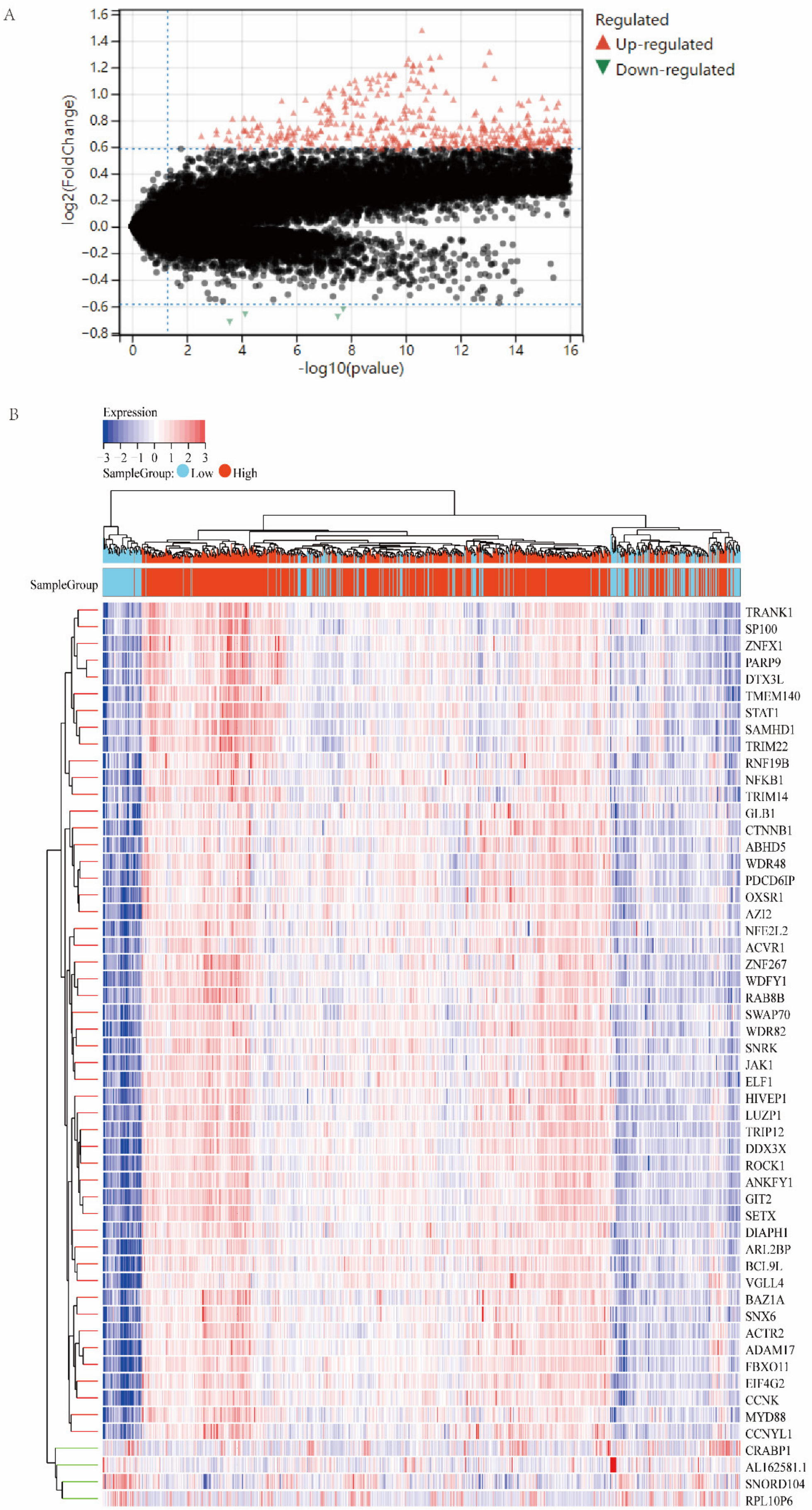
A
Regulated
Up-regulated
Down-regulated
log2(FoldChange)
-log10(pvalue)
B
Expression
SampleGroup: Low High
SampleGroup
TRANK1
SP100
ZNFX1
PARP9
DTX3L
TMEM140
STAT1
SAMHD1
TRIM22
RNF19B
NFKB1
TRIM14
GLB1
CTNNB1
ABHD5
WDR48
PDCD6IP
OXSR1
AZI2
NFE2L2
ACVR1
ZNF267
WDFY1
RAB8B
SWAP70
WDR82
SNRK
JAK1
ELF1
HIVEP1
LUZP1
TRIP12
DDX3X
ROCK1
ANKFY1
GIT2
SETX
DIAPH1
ARL2BP
BCL9L
VGLL4
BAZ1A
SNX6
ACTR2
ADAM17
FBXO11
EIF4G2
CCNK
MYD88
CCNYL1
CRABP1
AL162581.1
SNORD104
RPL10P6
